# Foreign Correspondence

**Published:** 1884-02

**Authors:** 


### Foreign Correspondence.

---

CHIHUAHUA, MEXICO, November 17, 1883.

TO THE CHICAGO MEDICAL JOURNAL AND EXAMINER:

This city of 20,000 souls, the capital of one of the Montezuman States, of unknown age, is situated among the "everlasting hills," that richly abound in ores of copper, gold, and silver; and, notwithstanding the encroachments of a higher civilization and more elegant designs in architecture, still preserves, intact, all the elements and characteristics of the typical Aztec *ciudad* of a hundred years ago, including the omnipresent one-story quadrangular adobe building, enclosing a green or paved plaza, which, among the wealthier, is often cooled by the fountain's spray, shaded by semi-tropical foliage, and perfumed by the breath of many flowers. There the family lives, shut out from the world by the immense carved wooden door overhanging the edge of the paved street, and by the iron grating of the windows seen everywhere, which give to the stranger, at first sight, the impression of being in the midst of a vast prison.

Many Americans are now coming in—many adventurers and many in search of health. The Mexican Central Railroad is now completed over 200 miles below here, and by April 1st, 1884, the gentlemanly officers inform me, it will be completed to the City of Mexico, a distance of 1,250 miles from Paso Del Norte. And as the climate in these parts, together with the scenery, affords the strongest allurements to those in quest of health, rest, and pleasure, it is not going too far to say that the cleanliness, smoothness, and dustlessness of this road, combined with elegant and marvellously clean stations and general offices, rob railway travel of its terrors. The cars are light and comfortable, and and move so smoothly that riding on the fastest trains is not irksome. The road in all its appointments is unique, and the civilizing influences of the North will surely but slowly follow it to its terminus. Here, at an elevation of about 4,000 feet, are not found extremes of temperature, nor "squalls" of weather. The thermometer will register from 30° to 108° F., with a mean annual temperature of 65°, and when the days are warmest the nights are always cool. Many at this season go lightly clad—barefooted; and to-day a six-year-old boy, by a neighboring brook where his mother was washing clothes with the soap weed, played joyfully in a state of perfect nudity. The atmosphere is dry and exceedingly light and exhilerating, and the appetite of everybody is good and readily satiated with fried quail, deer, antelope, etc., at the rate of $18 per week, *al casa Americana.* There is not the least doubt that the words of an old U. S. Army surgeon, of ten years residence in the country, are true: "When this country is better known, it will be the grandest resort on the American continent for invalids, as dyspeptic, consumptive, and neurasthemic patients and over-worked professional and business men, who can here, in a very short time, regain their health and recuperate their wasted energies." Here, on the plateaus, the epidemics of childhood, of the temperate regions, do not occur so frequently, nor are they so difficult of management, being less malignant. Diphtheria and scarlatina are rare. Isolated cases of small-pox occur frequently throughout Northern Mexico, yet the disease has been epidemic but once (6 years ago) in years. Vaccination is compulsory, and the Mexican Central road has now a physician vaccinating all the employes of the road, though no small-pox exists along the line. Yellow fever, so fatal on all the coasts, is unknown here, and the occurrence of true typhoid fever is rare, and when found is usnally in an American subject. Tape-worm is very common here and about Paso Del Norte, 225 miles north, and the cause is variously accounted for by competent medical observers. One of these, during the past two years, removed 17 worms, tinea soleum, from as many patients, "*every one* of whom was fond of and freely ate, rare-cooked native beef." Another assigned the cause to the frequent use of mutton, while a third one observed that the worm occurs most frequently among the peons and the poorer classes, who are in the habit of eating great numbers of rabbits. All may be right; but as rabbits are known to contain great numbers of cysticerci, there is no doubt that they are pre-eminently the cause of trouble in this country. The usual remedy is male fern and pumpkin seed. Of course, Mexico has been celebrated for the frequency of the venereal diseases, and, if one may judge by the pink scleroticas seen on every hand, and by the declaration of resident physicians that "Every other man you meet carries his own souud or catheter," she certainly deserves and maintains her unenviable notoriety.

"Stone in the bladder," said Dr. Frank Paschal, of this city, after eight years experience, "is quite common." The doctor is a very celebrated surgeon and lithotomist, and is justly entitled to his reputation, as he has operated for stone (mostly by the lateral, but sometimes by the median incision) seventeen times on patients, from 3 to 3½ years of age and up to 70 odd years, with but two fatal results, and these died from exhaustion, the operation having been too long delayed. Dr. Paschal informs me that the average Mexican will stand a capital surgical operation to which an American would quickly succumb, with little or no shock, and that, in so much, is the field of surgery widened here. In midwifery, the frequent use of the forceps is demanded, and yet uterine lacerations and displacements are not very frequent. Ovarian disease is rare throughout Mexico. Only sixteen ovariotomies have been performed in the county, and only two of them outside of the capital, and nearly every one proved fatal. Uterine cancer is exceedingly common, and its frequency is often charged up to the effect of a slightcervical laceration occurring in a syphilitic constitution. The University of Mexico dispenses the sciences of the capital in Spanish and French, and there are published in the whole country but three medical periodicals.

And now, lest something may have been said, unintentionally, whereby some young professional reader might be induced to seek a location and a national reputation like our most worthy and esteemed countryman, Dr. Paschal, it may be well enough to consider other things besides the following: And, 1st—The average Mexican has neither forgiven nor forgotten America's mean acts of 1846–7. His ways and thoughts are not your ways and thoughts. He is naturally suspicious, and though he may envy *los Americanos*, he is jealous of them and their inroads upon Mexican traditions, customs, and superstitions. "It takes two days to distribute one day's mail" in Los Coreos. Drugs are mostly imported and are enormously dear, being subjected to national, State, county, and municipal duties—the national duty alone increasing the cost 100 per cent. (nearly) over American prices. But now, if you will come, proceed thus: Send your diploma to the United States (American) Secretary of State, asking him to endorse it as genuine; then to your nearest Mexican Consul, who will likewise sign and send it to the Governor of the State you intend visiting, who, having examined, approved, and endorsed it, will send it for like action to the President of this Republic who may return it to you. You will now present yourself for examination, and not only prove a knowledge of *spoken* Spanish, but also a knowledge of the Mexican's everyday names of drugs, as well as their chemical and botanical names. The Board may examine in any or all the branches of medical science. Having been approved, you will then find some elegant senors, senoras, and senoritas, and take up your abode among a unique people, but in a magnificent country, full of grand scenery and grand possibilities, where invigorating climate will come to your rescue, enabling you to realize the effect on over-worked, broken-down constitutions of Dame Nature's magic restorative powers; for, after all, we must admit *en su poder nosotros podemos ganar la victoria.*

---

DR. DAVID W. YANDELL was recently elected an honorary member of the London Medical Society. We heartily congratulate the London Medical Society on its ability to secure so honored a name for its roster.

